# A kinase-dead knock-in mutation in mTOR leads to early embryonic lethality and is dispensable for the immune system in heterozygous mice

**DOI:** 10.1186/1471-2172-10-28

**Published:** 2009-05-20

**Authors:** Boris Shor, Druie Cavender, Crafford Harris

**Affiliations:** 1 Inflammation Research Team, Drug Discovery, Johnson and Johnson Pharmaceutical Research and Development, LLC, Raritan, NJ, USA; 2 Current address: Oncology Discovery, Wyeth Research, Pearl River, NY, USA; 3 Current address: Johnson and Johnson Skin Research Center, Skillman, NJ, USA

## Abstract

**Background:**

The mammalian target of rapamycin protein (mTOR) is an evolutionarily conserved kinase that regulates protein synthesis, cell cycle progression and proliferation in response to various environmental cues. As a critical downstream mediator of PI3K signaling, mTOR is important for lymphocyte development and function of mature T and B-cells. Most studies of mTOR in immune responses have relied on the use of pharmacological inhibitors, such as rapamycin. Rapamycin-FKBP12 complex exerts its immunosuppressive and anti-proliferative effect by binding outside the kinase domain of mTOR, and subsequently inhibiting downstream mTOR signaling.

**Results:**

To determine the requirement for mTOR kinase activity in the immune system function, we generated knock-in mice carrying a mutation (D2338) in the catalytic domain of mTOR. While homozygous mTOR kd/kd embryos died before embryonic day 6.5, heterozygous mTOR+/kd mice appeared entirely normal and are fertile. mTOR +/kd mice exhibited normal T and B cell development and unaltered proliferative responses of splenocytes to IL-2 and TCR/CD28. In addition, heterozygousity for the mTOR kinase-dead allele did not sensitize T cells to rapamycin in a CD3-mediated proliferation assay. Unexpectedly, mTOR kinase activity towards its substrate 4E-BP1 was not decreased in hearts and livers from heterozygous animals.

**Conclusion:**

Altogether, our findings indicate that mTOR kinase activity is indispensable for the early development of mouse embryos. Moreover, a single wild type mTOR allele is sufficient to maintain normal postnatal growth and lymphocyte development and proliferation.

## Background

The mammalian target of rapamycin (mTOR) is a serine-threonine kinase and a member of the phosphoinositide kinase related-kinase family (PIKK), which is evolutionary conserved from yeast to humans. mTOR acts as a sensor kinase that coordinates cellular response to growth factors, nutrients and energy availability in mammalian cells [1,2]. Natural product rapamycin, in complex with immunophilin FKBP12, binds the FKBP12-rapamycin binding (FRB) domain of mTOR and inhibits phosphorylation of downstream substrates 4E-BP1 and S6K1 [3]. One of the established roles of mTOR within the "rapamycin-sensitive" mTORC1 complex is to enhance translation rates though the direct phosphorylation of S6K1 and 4E-BP1 in response to mitogen and nutrient stimulation. Another, functionally distinct "rapamycin-insensitive" mTORC2 complex phosphorylates AKT and regulates cytoskeletal organization in yet understood fashion. [4]. To date, it is clear that mTOR signaling controls cell cycle progression, cell growth and proliferation by fine-tuning multiple metabolic circuits at the cell autonomous or organismal level. In many human cancers, deregulation of mTOR signaling, which is caused by the loss of critical tumor suppressors (PTEN, TSC1/2, LKB1), somatic mutations or gene amplifications of PI3CA (p110 alpha subunit of PI3K) or activating mutations in AKT, ultimately leads to increased cell growth, cell survival, and suppression of autophagy [5].

Previous studies of the in vivo functions of mTOR in adult metazoans were hampered by the early embryonic lethality or developmental arrest of TOR loss-of-function mutants. [6-9]. On the other hand, blocking mTOR with rapamycin, an agent that exhibits potent immunosuppressive efficacy in animal models and in clinics, provided important clues for the current understanding of mTOR function in immune responses. For example, rapamycin suppresses T cell proliferation in part through its inhibitory effects on cytokine production, cytokine signaling, and on T cell receptor/CD28 mediated lymphocyte activation [10]. The above effects are linked to the integral role of mTOR in control of G1- to S- phase of cell cycle. While the exact molecular mechanisms by which mTOR controls T cell division remain unknown, mTOR is responsible for activation of Cdk2 and Cdc2 kinases, downregulation of p27Kip1 and the induction of D-cyclins [11-13]. More recent study has demonstrated a direct physical association between mTOR, aurora B, S6K and 4E-BP1 that determines G1-S checkpoint in T cells [14]. Specifically, Aurora B and mTOR cross-regulate each other: rapamycin reduces aurora B kinase activity and aurora B – mediated events, such as Rb phosphorylation, induction of cyclin A and activation of Cdk1 and Cdk2 in primed T cells, whereas expression of aurora B enhances phosphorylation of S6K1 and 4E-BP1 [14]. Another report described a novel action of mTOR as a regulator of T cell migration during immune activation, where mTOR uniquely restricts the expression of L-selectin CD62L, chemokine receptor CCR7 and sphingosine 1-phosphate receptor type 1 (S1P_1_), presumably through the downregulation of the transcription factor KLF2 in activated T cells [15]. In addition to T cells, rapamycin can also interfere with B cell activation, proliferation and development, as well as with the function of mast cells [16-19].

Although mTOR is a downstream player of PI3K-Akt signaling pathway in numerous cell types, it can also respond to PI3K-independent signals, such as levels of amino acids or energy status. Indeed, cooperation between mTOR and PI3K signaling is important for proper regulation of lymphocyte size, metabolic activity, and cell cycle progression. Rapamycin and LY294002, a PI3K inhibitor, can target parallel pathways; combining LY294002 and rapamycin in human peripheral blood lymphocytes (PBL) stimulated by TCR or in human Kit225 cells induced by IL-2 leads to a synergystic suppression of proliferation. This correlates with differential effect of each drug on Cyclin D2 and Cyclin D3 expression [13]. Another example of where mTOR kinase can be stimulated in a PI3K-independent fashion is transformed B cell lines, in which induction of mTOR signaling, but not of PI3K or MEK, is sensitive to the withdrawal of nutrients [20].

Blocking mTOR signaling with rapamycin is largely explained by the inhibitory effect of the drug on mTORC1 complex. In this context, rapamycin acts as an allosteric mTOR inhibitor that does not bind the ATP binding pocket of mTOR kinase. However, the exact mechanism of how rapamycin inhibits mTOR signaling is not fully understood. One of the possibilities is that it blocks kinase-independent functions of mTOR by disrupting interactions between mTOR and its binding partners. Indeed, rapamycin is known to dissociate raptor-mTOR interactions resulting in attenuated recognition of substrates by mTOR [21] or to abolish phosphatidic acid binding to mTOR [22]. Additional, kinase-independent mechanism of rapamycin action is known in yeast, where rapamycin activates protein phosphotase 2A [23]. This and the emergence of mTORC2, a complex that is insensitive to acute rapamycin treatment, suggests a presence of previously unrecognized, mTOR kinase-dependent biological responses that are not blocked by rapamycin.

To understand the functional relevance of mTOR enzymatic activity in mouse development and in immune responses in vivo, we generated kinase-dead knock-in mutant mice. This strategy allowed us to avoid "secondary" effects that could be caused by conventional gene disruption and to ensure appropriate relative stoichiometry between mTOR and other components of mTORC1 and mTORC2 complexes. Our results reveal a critical role for mTOR kinase catalytic activity for the early embryonic development. Because of the lethality of homozygous mTOR knock-in embryos, we analyzed the lymphocyte development, proliferative responses and lymphocyte sensitivity to rapamycin in vitro in heterozygous animals.

## Results

### Generation of mTOR D2338A knock-in mice

**Figure 1:**
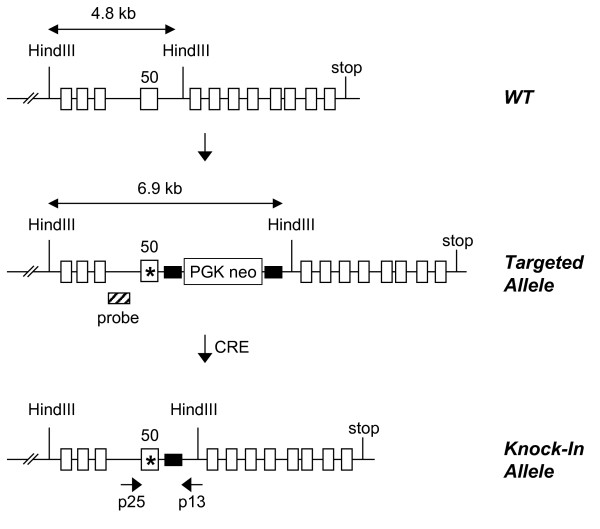
**Generation of mTOR kinase-dead knock-in mice**. Schematic map of the wild-type mTOR gene locus, targeted allele before and after Cre recombination. Exons are represented by white boxes. LoxP sites are shown as black boxes. Positions of Southern blot probes, PCR primers, and restriction enzyme sites are indicated. PGK-Neo, neomycin resistance gene. Asterisk shows D2338A mutation in exon 50.

**Figure 2:**
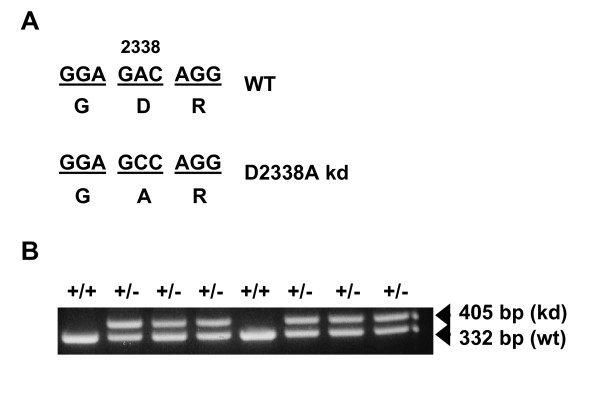
**mTOR kinase-dead allele and its confirmation in mice**. (A) Nucleotide and corresponding amino acid sequence of the wild type or mutated allele. (B) PCR-based genotyping using genomic DNA and a primer set (p13 and p25) flanking the remaining loxP site. The 332 bp band corresponds to the wild type allele, while 405 bp band corresponds to the mutant allele containing the remaining loxP site.

To understand the in vivo role of the mTOR kinase catalytic activity, we set out to generate knock-in mice with a targeted replacement of the wild type mTOR allele with a mutant version in which Asp 2338 was changed to Ala (D2338A) (Fig 1, Methods). This D2338A substitution (Fig. 2A) is well known to eliminate phosphotransferase activity in vitro and suppress mTOR signaling in cell cultures or in vivo [24-28]. Correctly targeted ES clones harboring a mutant mTOR allele with PGK-Neo cassette were identified by Southern blotting with an appropriate probe (see Additional file 1). Genotyping of the heterozygous mTOR kinase-dead (mTOR+/kd) mice was achieved by PCR analysis of genomic DNA. As expected, approximately 332-bp and 405-bp fragments were obtained from the wild-type and kinase-dead mutant alleles, respectively (Fig. 2B, Methods). Presence of D2338A mutation in the offspring mice was further confirmed by conventional sequencing of the amplified fragments or by pyrosequencing (data not shown).

### Embryonic lethality of mTOR homozygous kinase-dead knock-in mice

Intercrossing of heterozygous animals followed by PCR genotyping of the offspring revealed that no homozygous pups can be recovered at birth. Wild-type and heterozygous mice however, were born at the expected 1:2 ratio (of the 101 first-generation mice, 29 were +/+ and 72 were +/kd, Table 1), indicating that mTOR kinase-dead mutation is recessive embryonic lethal. Next, to determine the time of embryonic lethality, embryos were collected at different stages of gestation. Strikingly, no homozygous mTOR kd/kd embryos were detected at embryonic day 16.5 (E16.5), 13.5, 12.5, 8.5, or 6.5 (Table 1). These findings demonstrate that mTOR kinase-dead embryos die within E6.6 stage and are consistent with a role of mTOR in early embryonic development.

**Table 1:** Genotypes of offspring from intercrosses of mTOR+/kd mice.

	**Genotype**				
Age	+/+	+/kd	kd/kd	Resorbed	Total
E6.5	2	7	0	0	9
E8.5	1	8	0	0	9
E12.5	3	6	0	0	9
E13.5	2	19	0	0	21
E16.5	4	8	0	3	12
3 weeks	29	72	0	N/A	101

N/A, not applicable

Genotyping of embryos was performed by PCR as in Fig. 2B. Number of embryos with corresponding genotypes is shown.

### General phenotype of mice heterozygous for the mTOR knock-in allele

**Figure 3:**
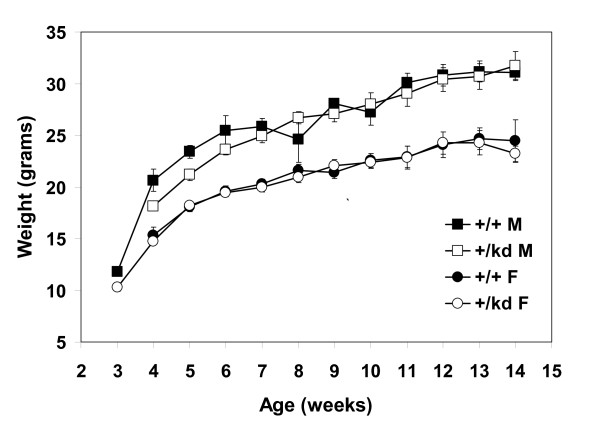
**Average body weights of mTOR+/kd knock-in mice**. Shown are growth curves of female and male mTOR+/+ or mTOR+/kd animals. Values are means ± S.E.M., n = 4–15. F, female; M, male.

Heterozygous mTOR kinase-dead mice were fertile and healthy. It was previously reported that heterozygous PI3K/p110α kinase-dead knock-in mice and S6K1 knock-out mice exhibit reduction in body weight [29,30]. Therefore, we compared body weights among mTOR+/+ and mTOR+/kd mice during postnatal growth. There was no significant difference in body weights or pattern of growth, organ size, gross appearance or behavior observed between wild type and heterozygous mice at least up to 14 months of age (Fig. 3; data not shown). Although heterozygous male mice were slightly smaller during weeks 4–6, the average body weights were not statistically different from those of wild type males (p > 0.05).

### mTOR mRNA and protein expression in mTOR heterozygous kinase-dead knock-in mice

**Figure 4:**
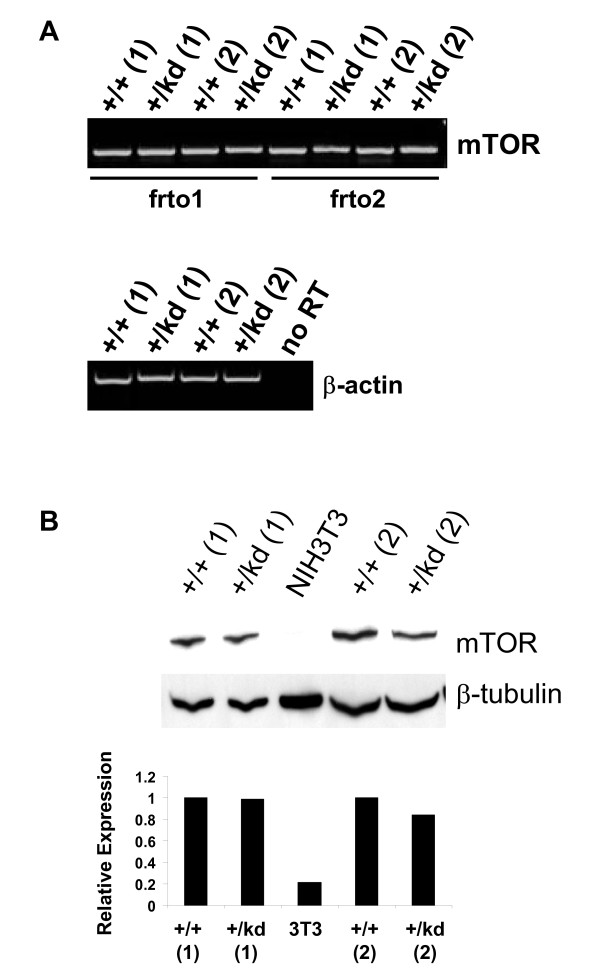
**Expression of mTOR in wild type and +/kd knock-in mice**. (A) RT-PCR analysis of mRNA from hearts of wild-type and mTOR+/kd mice using two different pairs of primers frto1 and frto2. β-actin is used as a positive control. (B) Immunoblot of mTOR in protein extracts (10 μg) from heart tissues of mTOR+/+ and mTOR+/kd mice and its semiquantification by densitometry analysis. β-tubulin is loading control. Two independent pairs of +/+ and +/kd mice are shown.

RT-PCR was used to determine the transcript levels of mTOR in heterozygous mTOR kinase-dead vs. wild-type mice. mTOR mRNA expression was analyzed in the heart tissues from two pairs of mTOR+/+ and mTOR+/kd animals using two different primer sets. Similar levels of mTOR mRNA were detected in both wild-type and heterozygous kinase-dead mice (Fig. 4A), indicating that the presence of point mutation and of the remaining loxP site in the downstream intron does not affect mTOR mRNA expression in the heterozygous mice. We also compared mTOR protein expression in the heart tissues of mTOR+/+ and mTOR+/kd mice by Westtern immunoblot. Quantification of mTOR protein expression levels from two pairs of mTOR+/+ and mTOR+/kd mice did not reveal any significant difference between two genotypes (Fig. 4B). These results indicate that mTOR heterozygous kinase-dead animals express full-length mTOR protein at normal levels and are consistent with our RT-PCR data.

### Immunological phenotype of mTOR kinase-dead heterozygous mice

**Figure 5:**
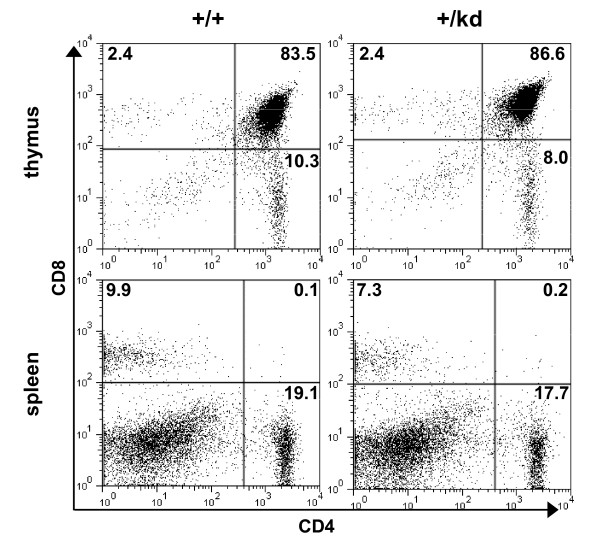
**Analysis of T cell populations in mTOR+/kd mice**. Total cells from thymi and spleens were analyzed by flow cytometry with the indicated antibodies, as described in Methods. All plots show cells in the lympchocyte gate. Numbers indicate the percentage of gated cells in particular quadrant. The data are representative of three pairs of mice examined. Thymocytes and splenocytes were stained for CD4 and CD8.

**Figure 6:**
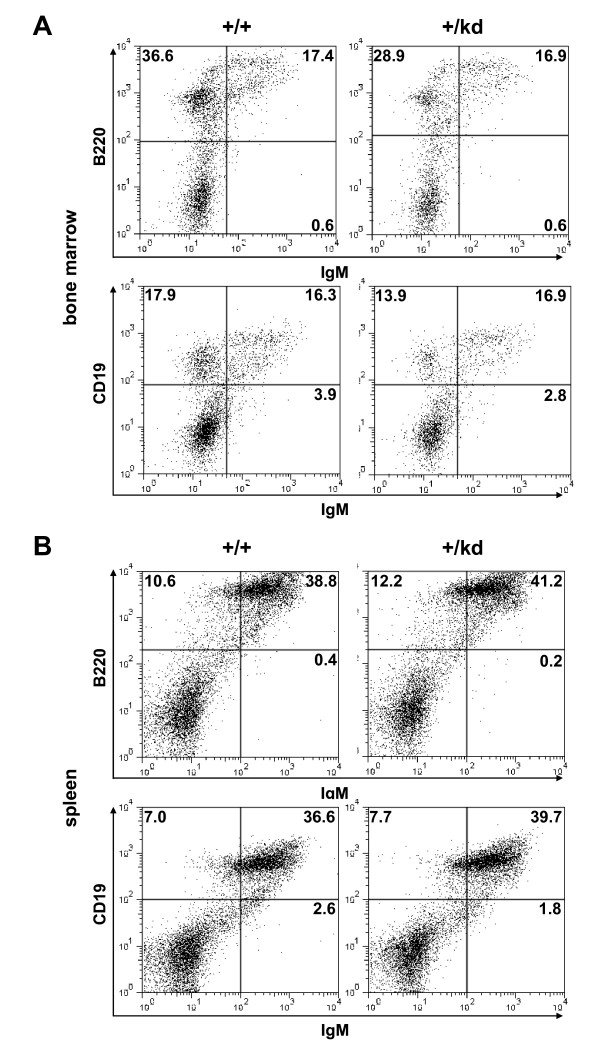
**Analysis of B cell populations in mTOR+/kd mice**. Total cells from spleens and bone marrows were analyzed by flow cytometry as described in Methods. All plots show cells in the lympchocyte gate. Numbers indicate the percentage of gated cells in particular quadrant. The data are representative of three pairs of mice examined. (A) Bone marrow cells were stained for immunoglobulin M (IgM) and B220 (*top*) or IgM and CD19 (*bottom*). (B) Splenocytes were stained for IgM and B220 (*top*) or IgM and CD19 (*bottom*).

Given the inhibitory effects of rapamycin on T- and B-cell mediated immune responses, we analyzed relative distribution of immune cell subsets in thymus, spleen, and bone marrow of mTOR+/+ and mTOR+/kd littermates. Flow cytometric analysis revealed no differences between mTOR+/+ and mTOR+/kd mice in the percentages of double negative, double positive and single positive CD4 and CD8 expressing cells in the thymus (Fig. 5). In the periphery, the proportions of splenic CD4 and CD8 T cells were also comparable. Moreover, we did not observe significant changes in the number of T cells detected with CD3 antibody in thymi and spleens of mTOR+/kd mice (see Additional file 2). Collectively, these findings indicate that T cell maturation in thymus or periphery is not perturbed in mTOR+/kd mice. We also investigated B cell development in mTOR heterozygous mutant mice. The levels of more mature IgM+ B220+ and IgM+ CD19+ B cells in the bone marrow were comparable in mTOR+/+ and TOR+/kd littermates (Fig 6A). However, the bone marrow of mTOR+/kd mice had slightly reduced fraction of IgM- B220+ and IgM- CD19+ B cells. Finally, our analysis revealed no difference in the proportions of B-lymphocyte populations in the spleens of wild-type and mTOR+/kd mice (Fig. 6B). These results show that presence of a single functional mTOR allele is sufficient to support the T- and B-cell development in mice.

### Mature T cell proliferation

**Figure 7:**
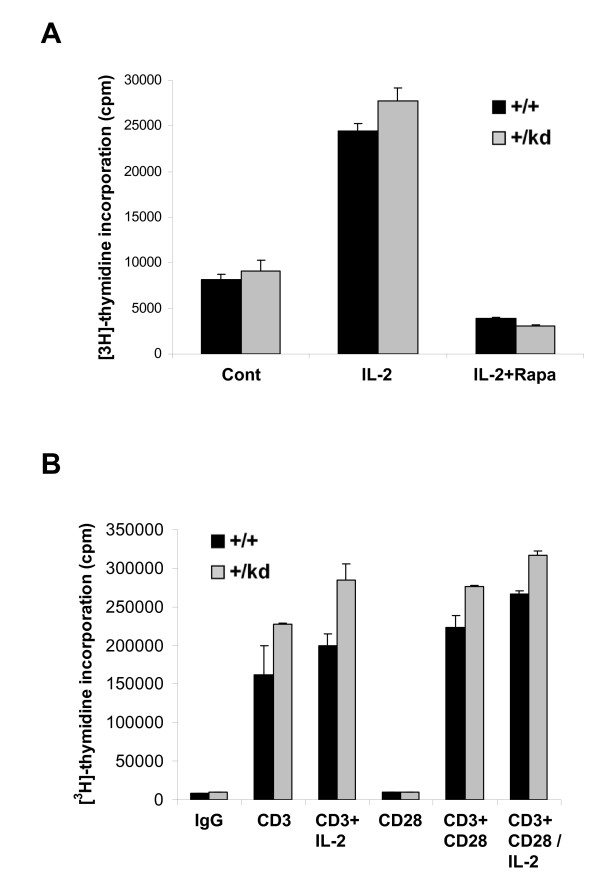
**Proliferative capacity of mTOR+/kd T cells**. (A) Proliferation of splenocytes after IL-2 stimulation. Spleen cells from mTOR+/+ and mTOR+/kd mice were stimulated with IL-2 in the presence or absence of rapamycin and proliferation was assessed by [^3^H] thymidine incorporation, as described in Methods. (B) Proliferation of spleen cells from mTOR+/+ and mTOR+/kd littermates in response to anti-CD3 with or without soluble anti-CD28 or IL-2 was measured as in (A). Data are expressed as % control. Results shown are representative of three experiments.

**Figure 8:**
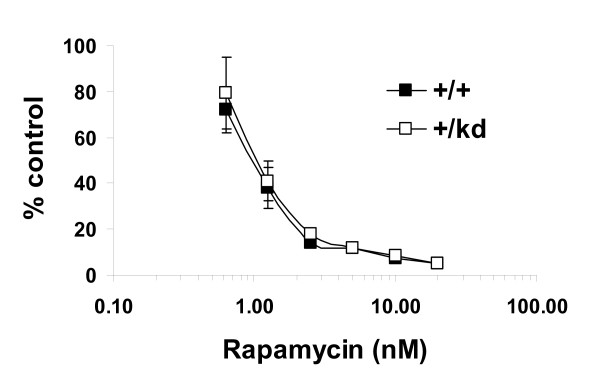
**Response of mTOR+/kd T cells to rapamycin**. Purified T cells from spleens of mTOR+/+ and mTOR+/kd mice were cultured on anti-CD3-bound plates in the presence of various doses of rapamycin and proliferation was measured as in Fig 7. Data are expressed as % control. Results shown are representative of three experiments.

Previous reports have demonstrated that rapamycin suppresses IL-2- and TCR/CD28-driven cell proliferation and G1- to S-phase progression of T-cells. Furthermore, lowering TOR levels with siRNA or genetically is known to cause hypersensitivity to rapamycin in model organisms and in vitro [8,31,32]. Specifically, growth of Drosophila larvae that are heterozygous for dTOR was more profoundly delayed in the presence of rapamycin compared to the wild-type controls [8,31,32]. Therefore, we first investigated proliferative responses of lymphocytes from mTOR+/kd mice to various T cell stimuli. Proliferation of splenic T cells in response to IL-2 stimulation, anti-CD3 stimulation alone or anti-CD3 antibody in combination with IL-2 or with anti-CD28 was not impaired in mTOR+/kd compared to wild-type mice (Fig. 7A and 7B). As expected, treatment with 30 nM rapamycin potently inhibited IL-2-driven proliferation of wild-type splenocytes (Fig. 7A). Similar sensitivity to rapamycin was also observed for the lymphocytes from mTOR+/kd mice. Next, inhibitory effects of rapamycin on anti-CD3-induced proliferation of wild-type and heterozygous cells were compared using purified T cells grown in the presence or absence of different concentrations of the drug. The proliferative response of T cells to anti-CD3 stimulation was equally well inhibited by rapamycin with IC_50 _values of ~1 nM for cells of both genotypes (Fig. 8). These results indicate that T cell proliferation is normal in mTOR+/kd mouse and that a heterozygousity for a kinase-dead mTOR does not sensitize T cells to rapamycin in vitro.

### mTOR Kinase activity is normal in mTOR+/kd mice

**Figure 9:**
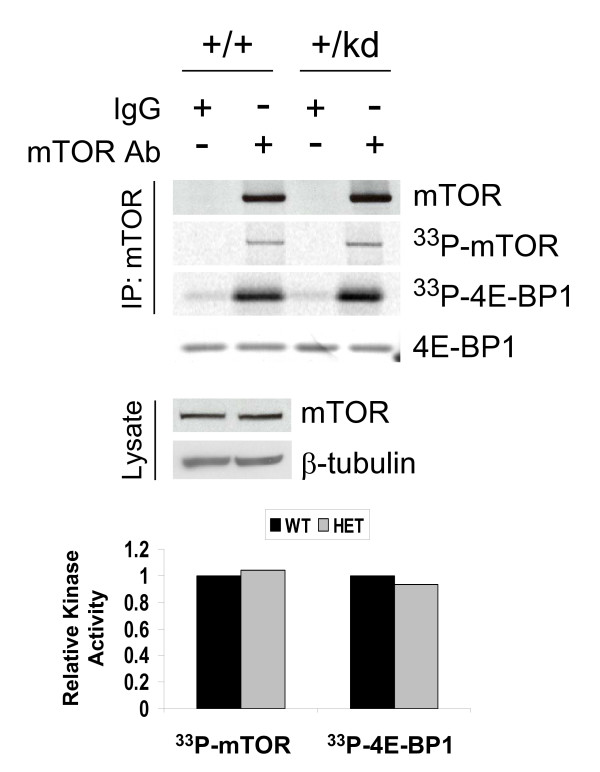
**Normal mTOR activity in mTOR+/kd mice**. mTOR was immunoprecipitated from heart lysates and the immune complex kinase assays were performed with 4E-BP1 as exogenous substrate, as described in Methods. The degree of phoshorylation of 4E-BP1 and mTOR was visualized via autoradiogram and quantified using a phosphoimager (*bottom*). Amount of mTOR in immunoprecipitates or in total lysates was assessed by immunoblotting with mTOR antibody. β-tubulin is used as a loading control. Equal amount of 4E-BP1 in the kinase reaction mixture was visualized by Ponceau S staining. Data are representative of two experiments.

Because only one catalytically active mTOR allele is present in all tissues from the kinase-dead heterozygous animals, we expected to observe nearly 50% loss in total mTOR kinase activity. Immunoprecipitation of the mTOR from heart and liver lysates of mTOR+/kd mice showed no change in kinase activity, as measured by phosphorylation of exogenously added 4E-BP1 and by auto-phoshorylation of the mTOR itself (Fig. 9, see Additional file 3). This finding suggests that there may be a compensatory up-regulation of mTOR activity in mTOR+/kd mice, as the total expression of mTOR remains unchanged (see Fig. 4).

## Discussion

Current understanding of the in vivo role of mTOR in the immune responses stems from the use of pharmacological inhibitors of the PI3-kinase signaling and nutrient-sensing mTOR pathway. The mTOR signaling inhibitor rapamycin is a widely used immunosuppressive drug that interferes with the lymphocyte function at multiple levels. It acts in part, by inhibiting T- and B-cell proliferation, T lymphocyte migration, or by promoting Treg cell differentiation [15,16,33-35]. Cancer biology has generated significant body of evidence indicating that rapamycin's effects on mTOR kinase activity are incomplete or are at times even different from the direct inactivation of kinase activity by mutations [3,36]. Furthermore, the deletion of mTOR gene in mice results in more pronounced phenotypes than those induced by rapamycin [6,7,37]. To avoid "secondary" effects caused by gene disruption we attempted to generate mTOR kinase-dead knock-in mice that will reflect the action of small molecule inhibitors more accurately. Unexpectedly, we found that a single site mutation in the mTOR kinase domain, a replacement of aspartic acid at position 2338 with alanine is essential for the early development of homozygous mouse embryos. As demonstrated in multiple studies, D2338A change renders mTOR kinase catalytically inactive, suggesting that observed embryonic lethality is a direct consequence of the loss of the mTOR kinase activity in vivo.

Similar to our report, early embryonic lethality was described for mTOR null mice with complete or partial (C-terminus) disruption of the mTOR gene [6,7]. Lethality of homozygous mTOR null embryos in these studies was linked to the defect in inner cell mass (ICM) and trophoblast proliferation of the blastocysts. Although we have not determined the exact reason and the timing of embryonic lethality in mTOR kd/kd mice, the fact that no homozygous embryos can be recovered at E6.5 in our study agrees well with developmental arrest of homozygous mTOR null embryos at E5.5 [7]. The reason why mTOR kd/kd mutant embryos cannot be detected at the same stage when homozygous mTOR null embryos are still present (E6.5 and E7.5), could be explained by the possible contamination of the mTOR kd/kd embryos with maternal tissue during the dissection procedure. Another example underscoring the role of mTOR in embryonic development is the ethylnitrosourea-induced "flat-top" mutation in the intron of mTOR [37]. The homozygous flat-top mouse mutant embryos obtained during the above study, however, survive to the much later stage (E12.5) than mTOR kd/kd mice, presumably due to the residual expression of the wild-type mTOR protein sufficient to support the embryonic development at earlier stages. In addition, profound defects in forebrain development in both flat-top embryos and rapamycin-induced phenocopy [38] were not observed in mTOR+/kd mice in our study.

mTOR+/kd knock-in mice showed no gross morphological or behavioral changes. This result is consistent with the phenotypes of homozygous mTOR null mice, showing no obvious anatomical defects. Furthermore, mTOR+/kd pups exhibited normal body weights, which is also in agreement with unaltered body sizes of heterozygous mTOR null mice. The ability of mTOR+/kd knock-in mice to progress through embryonic development and postnatal growth without obvious abnormalities suggests that a single remaining wild-type mTOR allele is sufficient to support normal mouse development. Interestingly, this is in contrast to the phenotypes of mice heterozygous for the kinase-dead knock-in mutation in PI3K p110α, which show growth retardation linked to the decrease in skeletal muscle mass [30].

Generation of the mTOR kinase-dead knock-in mice ensured appropriate relative stoichiometry between mTOR and other components of mTORC1 and mTORC2 complexes. For example, complete elimination of mTOR expression in homozygous mTOR null mice leads to more severe phenotype compared to the partial reduction in the functional mTOR protein in the flat-top mutants. Because mTOR+/kd mice contain one copy of wild-type allele and one copy of kinase-dead D2338A allele, they are expected to express normal levels of mTOR mRNA or a "full-length" mTOR protein. While we were not able to distinguish the product of the mutant allele from the wild type mTOR, our knock-in procedure did not interfere with either mTOR mRNA level or the total amount of mTOR protein in tissues from mTOR+/kd mice. Thus, an indispensable role of mTOR in early embryogenesis is likely due to the loss of its catalytic activity, rather than a disruption of mTOR containing protein complexes or a scaffolding function of the mTOR, both of which cannot be ruled out from the studies involving mTOR null or flat-top mutants. Given the established effects of rapamycin in T and B cell development, we examined the role of mTOR in the immune system in mice heterozygous for the kinase-dead mutation of mTOR. Surprisingly, FACS analysis of lymphoid compartments did not reveal any significant changes in T cell development in thymus or in generation and maintenance of mature T cells in the periphery of mTOR+/kd mice. While we have observed somewhat decreased frequency of IgM-B220+ and IgM-CD19+ subsets in the bone marrow, the precise stage of B cell development at which this reduction occurs awaits further investigation. Furthermore, the frequency of the more mature B cell subsets in the bone marrow and spleen was comparable between +/kd mice and their wild type littermates, suggesting that the development and maintenance of mature B cells were largely unperturbed in mTOR+/kd mice. Multiple studies utilizing rapamycin have also demonstrated a critical role for mTOR in the response of T cells to TCR/CD28 initiated stimuli or IL-2 driven cell expansion. In the present report, however, we found that IL-2 or CD3-mediated proliferative responses of T cells were not compromised in mTOR+/kd mice.

Reduction of TOR gene dosage in other organisms is known to cause hypersensitivity to rapamycin. For example, heterozygous Drosophila dTOR larvae are much more sensitive to rapamycin than wild type controls [8]. Combination of mTOR siRNA and rapamycin had also synergistic effect at inhibiting growth of several human cancer cell lines [31,32]. Thus, we hypothesized that proliferation of T cells isolated from mTOR+/kd animals could be more sensitive to the effects of the drug. Our finding that wild type T cell proliferation in response to anti-CD3 was inhibited by rapamycin in a dose-dependent manner with an IC50 value of 1 nM is consistent with previous reports [39,40]. Notably, proliferation of T cells of both genotypes was similarly inhibited by the drug at all doses when stimulated with CD3 anitbody or by 30 nM rapamycin when IL-2 was present in the medium. In addition to comparable proliferative responses between +/kd and wild type T cells, we found no significant alterations in the cell cycle profile of +/kd thymocytes (see Additional file 4). Thus, our data suggest that eliminating one functional allele of mTOR does no affect immune system function in mice.

There are two possible explanations of why mTOR+/kd mice did not show significant phenotypes compared to with wild type littermates. First, is that expected 50% reduction in total mTOR kinase activity is not sufficient to disrupt mTOR-related functions in mTOR+/kd mice. Second, it is possible that there could be a compensatory increase in catalytic activity of the protein generated from the wild type allele to account for the loss of kinase activity by the kinase-dead protein. Our data show that mTOR+/kd mice exhibit normal overall mTOR kinase activity in at least heart and liver tissues. While more accurate measurements of the total mTOR kinase activity and phosphorylation of the downstream mTOR effectors 4E-BP1 and S6K1 may be necessary, we think that compensatory upregulation of the mTOR kinase activity in a heterozygous setting is a possibility in at least some tissues. Although no previous knock-out studies addressed a role for the mTOR in the immune system function, mice heterozygous for mTOR did not exhibit any obvious alterations in morphology or growth, which is consistent with our findings. Interestingly, Drosphila larvae heterozygous for dTOR also grow at a similar rate as wild type controls [8].

## Conclusion

In summary, generation of mTOR kinase-dead knock-in mice demonstrated a critical role for mTOR catalytic activity in normal embryonic development. Since mTOR+/kd mice are indistinguishable from wild type in phenotypes, further studies involving conditional tissue-specific inactivation of mTOR will be necessary to clarify the role of mTOR in immune system and in diseases such as cancer.

## Methods

### Targeting vector and chimeric mouse production

mTOR kinase-dead knock-in heterozygous mice were developed in collaboration with Lexicon Genetics (Woodlands, TX) using a proprietary knock-in strategy. Briefly, to create the mTOR kinase-dead mutation, Asp 2338 was mutated to Ala in the pKOS28 genomic clone containing region of the wild type mTOR allele. The kinase-dead knock-in targeting vector was made by inserting a PGK Neo cassette flanked by 2 LoxP sites 26 nt after the exon 50. The targeting vector was transfected into Lex-1 ES cells (male) derived from the 129/SvEvBrd strain. Targeted ES cell clones were grafted into embryos and embryos were further implanted into female foster mice to generate chimeras. The PGK-Neo cassette was eliminated using Cre recombinase transgene during spermatogenesis in chimeric males, leaving only loxP junction sequence in the intron 50. Chimeric transmitting males were crossed into C57BL/6 (albino) females to generate heterozygotes bearing the point-mutated alleles. All experimental studies in mice were approved by the animal care and user committee of Johnson & Johnson Pharmaceutical Research and Development.

### Genotyping of targeted ES cells, mice, embryos

Homologous recombination in targeted ES cell clones was confirmed by Southern blot analysis. Genomic DNA was digested with HindIII and hybridized to the 5' probe. mTOR+/kd clones produced a 4.8 kb wild-type band and a 6.9 kb targeted band containing PGK Neo cassette (see Additional file 1). The genomic DNA of the offspring was extracted from their tails by using DNEasy kit (Qiagen). A set of two primers was used to amplify regions of genomic DNA present in either wild type or knock-in animals. PCR with the forward primer in intron 49 (p25; 5'-CTGTCACATGTGCTCTGGTG-3') and the reverse primer in intron 50 (p13; 5'-CTGTCATCTTAGCTCAGTGATG-3') generates a 332 bp fragment corresponding to a wild-type allele and a 405 bp fragment specific for the mutant mTOR-kd allele. To confirm the presence of the kinase-dead single nucleotide change mutations in the offspring mice, gel – purified PCR products were cloned into pcr2.1-TA vector (TOPO TA Cloning Kit; Invitrogen, CA) according to the manufacturer's instructions, and 10 independent clones were sequenced. Pyrosequencing and sample preparation was performed with PSQ 96MA analyzer in accordance with the manufacturer's instructions (Biotage, Inc). Genomic DNA was amplified with p25 and p13 (biotinylated at 5' position) primers. The pyrosequencing primer (PyrSeqF1; ACATTTTAGGCCTTGG) was complementary to the 16 bp common region 2 nucleotide upstream of the mutation site. Embryo genotyping was performed by PCR analysis of DNA isolated from either the yolk sac or the whole embryos.

### Body Weight

Body weight was measured with electronic balance at the indicated age. Because of the natural difference in body weight between males and females, each gender was analyzed separately. Data were expressed as mean ± SEM, n ≥ 5 for all groups at all ages.

### Flow Cytometric Analysis

mTOR+/kd mice were analyzed in parallel with age-matched wild type siblings. Single cell suspensions from the thymus and spleen were prepared by homogenization of the organs with the blunt end of a syringe plunger and were passed through a 70 μm nylon mesh cell strainer. Bone marrow cells were obtained from femurs and tibias by flushing with PBS using a 27-gauge needle and were filtered through a nylon mesh. Splenic cells were depleted of erythrocytes by osmotic lysis with 1XRBC lysis buffer (eBioscience) according to manufacturer instructions. Cell suspensions were washed once with 50 mL of staining buffer (eBioscience), centrifuged for 4 minutes at 4°C (400 g) and resuspended in staining buffer at 2 × 10^7 ^per ml. Prior to staining, cells were pre-incubated with anti-CD16/CD32 antibody cocktail (BD Biosciences) to block nonspecific Fc binding. Aliquots of 10^6 ^cells were stained with 1 μg of anti-CD4, -CD8, -CD3, -CD19, -IgM, or CD45R/B220 (BD Bioscences) at 4°C for 20 minutes. Monoclonal antibodies conjugated with phycoerythrin (PE), fluorescein isothiocyanate (FITC), or cychrome (CyC) were used in two- or three-color analyses. Flow cytometry was performed on a FACScan flow cytometer (BD Biosciences) and analyzed by using WinList 5.0 (Verity Software House) or FloJo (TreeStar) software. Cell cycle analysis of thymocytes was performed by staining with propidium iodide (BD Biosciences) according to the manufacturer's protocol. Cell cycle phase distribution was determined using ModFit (Verity Software House).

### Lymphocyte Isolation and Proliferation Assays

Mouse spleens were extracted under sterile conditions. Splenocyte cell suspensions were prepared as described above. Following red blood cell lysis, splenocytes were washed in PBS, and resuspended at 2 × 10^6 ^per ml in RPMI-1640 medium (Cellgro) containing 25 mM HEPES, L-Glutamine, 10% heat-inactivated fetal calf serum, antibiotic-antimycotic solution (100×, Gibco), and 2-mercaptoethanol (1000×, Gibco). 2 × 10^5 ^cells were plated in 96-well flat-bottom plates (Corning). Cells were further stimulated for 72 h with or without recombinant human IL-2 (10 U/well, R&D Systems), plate-bound anti-CD3 MAb (BD Biosciences, pre-coated at 2 μg/ml), 10 μg/ml anti-CD28 MAb (BD Biosciences) or IgG. When indicated, 10 μl of the medium containing either DMSO or rapamycin (Calbiochem, EMD Biosciences) was added prior to the stimulation. For the last 8 hours, cultures were pulsed with 1 μCi per well [^3^H] thymidine (GE Healthcare). Proliferation was measured using a FilterMate Cell Harvester (PerkinElmer) and counted with a TopCount NXT counter (PerkinElmer). All results are expressed as means ± standard error of triplicate cultures. For rapamycin titration experiments, splenic T cells were purified from total splenocytes with negative selection antibody mix (CD11b, Ter-119, CD21, CD45R/B220, CD19), followed by incubation with magnetic beads (Dynal, Invitrogen) according to the manufacturer's specifications. Purified cells were plated in commercially available anti-CD3 coated 96-well T cell activation plates (BD Biosciences) and proliferation was assessed by the incorporation of [^3^H] thymidine as described above.

### Western blot analysis, Immunoprecipitation, kinase assay

Organs of interest were excised immediately after sacrificing the animals and processed right away or stored at -80°C until further analysis. Tissues were homogenized using Ultra-Turrax T8 homogenizer in 3 ml ice-cold lysis buffer containing 50 mM Tris-HCl pH7.4, 100 mM NaCl, 50 mM β-glycerolphosphate, 10% glycerol, 1 mM DTT, 1 mM MgCl2, 1 mM PMSF, Phoshatase Inhibitor Cocktail 1 and 2 (Sigma) and complete EDTA-free protease inhibitors (Roche Applied Science). The cell lysates were centrifuged twice for 15 min at 12,000 × g at 4°C to remove cellular debris. Protein extracts were further used to detect total mTOR level or for immunoprecipitation. mTOR was immunoprecipitated from 1.5 ml cleared cell lysates by incubation with 12 μl of anti-mTOR antibody, mTab1 (Upstate, Millipore) for 12 hours at 4°C, followed by incubation with 100 μl of 50% washed recombinant protein G agarose beads (Invitrogen) for an additional 4 hours. Immunoprecipitation with control IgG was performed in parallel. The agarose beads were washed twice with 1 ml of ice-cold wash buffer (lysis buffer containing 1% w/v Tween 20), once with 1 ml of high salt buffer (100 mM Tris-HCl pH7.4, 500 mM LiCl), and twice with 1 ml of kinase buffer A (10 mM HEPES pH7.4, 50 mM NaCl). To assay kinase activity, the immunocomplexed beads were resuspended in 25 μl of kinase buffer B containing kinase buffer A, 1 mM DTT, Phoshatase Inhibitor Cocktail 1 and 2 (Sigma), complete EDTA-free protease inhibitors (Roche Applied Science), 50 mM β-glycerolphosphate, 2 mM EDTA. 25 μl of the reaction mixture [kinase buffer B, 100 μM ATP, 20 mM MnCl_2_, 20 mM MgCl_2_, 0.5 μg 4E-BP1 (Stratagene), and 10 μCi [γ-^33^P]ATP] was combined with beads and incubated at 30°C for 30 min, followed by termination with 15 μl of 4× LDS sample buffer (Invitrogen). Reaction products were subjected to 4–12% SDS-PAGE and transferred to the nitrocellulose membrane according to manufacturer instructions (Invitrogen). Incorporation of ^33^P into 4E-BP1 was analyzed by PhosphoImager (Molecular Dynamics). For relative quantification of mTOR kinase activity, incorporation values were normalized to the amount of total mTOR present in each reaction mixture determined via densitometry of mTOR immunoblots. When desired, the transferred proteins were visualized by Ponceau S staining.

To detect mTOR, blots were probed with 1:500 dillution of rabbit mTab1 antibody following the manufacturer's protocol (Upstate, Millipore). For tubulin detection, a 1:1000 dilution of mouse anti-β-tubulin antibody (Upstate, Millipore) was used. The secondary antibody was a 1:10,000 dilution of goat or mouse anti-rabbit IgG horseradish peroxidase (HRP) conjugate (Pierce, Thermo Scientific). Immunoblots were detected using the ECL Plus system (GE Life Sciences). Extracts from serum treated NIH3T3 cells (Cell Signaling) were used as a control to visualize correct position of mTOR band on a western blot.

### RNA Isolation and RT-PCR Analysis

For total RNA isolation, mouse heart tissues were cut into slices and stabilized in RNAlater RNA Stabilization Reagent (Qiagen) for 1 week. Next, tissues were homogenized using Ultra-Turrax T8 homogenizer and total RNA was isolated with RNeasy Protect Mini Kit (Qiagen). To digest DNA, samples were treated with DNA-free DNAse (Ambion). The OneStep RT-PCR kit (Qiagen) was used to quantitate RNA. RNAse Inhibitor (SUPERase In, Ambion) was included in all RT-PCR reactions. RT-PCR was performed using specific oligonucleotide primers spanning two different regions of mTOR mRNA. Primers were designed so that generation of 353-bp products is a result of mRNA amplification, ruling out possibility for genomic DNA contamination. Mouse β-actin was used as a control amplicon to assess constitutive transcription rates. The sequences of primers used for the detection of mTOR mRNA are as follows: forward fto1 5'-AACAACACAGCTGGGGACGA-3', reverse rto1 5'-TCTCGGAGCACTTCCATCACA-3'; forward fto2 5'-AAGGCCTGATGGGATTTGG-3', reverse rto2 5'-TGTCAAGTACACGGGGCAAG-3'. β-actin primer set (forward 5'-TGTGATGGTGGGAATGGGTCAG-3', reverse 5'-TTTGATGTCACGCACGATTTCC-3') was from Stratagene. The conditions were dependent upon Tm of the primers and initial amount of template. The amplification parameters were the following. Reverse transcription at 50°C for 30 min, PCR initial activation at 95°C for 15 min, denaturation at 94°C for 30 s, annealing at 50–57°C for 20 s, extension at 72°C for 30 s.in a 35 cycle program.

## Abbreviations

CCI-779: cell cycle inhibitor-779; DMSO: dimethylsulfoxide; DTT: dithiothreitol; E: embryonic stage; ES: embryonic stem; FK506: Tacrolimus; FKBP12: FK506 binding protein 12; FRB: FKBP12-rapamycin binding domain; FRB: FKBP12-Rapamycin Binding; IC_50_: the half maximal inhibitory concentration; kd: kinase-dead; mTOR: mammalian target of rapamycin; mTORC: mTOR complex; PBL: peripheral blood lymphocytes; PI3K: phosphoinositide 3-kinases; PIKK: phosphoinositide-3-kinase-related kinase.

## Competing interests

The authors declare that they have no competing interests.

## Authors' contributions

BS carried out the experiments and drafted the manuscript. DC is responsible for overall project management and proofreading of the manuscript. CH conceived of the study, and participated in its design and coordination. All authors read and approved the final manuscript.

## Supplementary Material

Additional file 1 **Southern blot verification of targeted ES cell clones**. Hybridization of Hind III digested genomic DNA from ES cells probed with a 5' external probe shows correct targeting event in lanes#1–3. Lane 4 is a control DNA from parental cells. Click here for file

Additional file 2 **CD3 expression in the thymocytes of mTOR+/kd mice**. Thymocytes from mTOR+/+ and mTOR+/kd mice were stained for CD3 and analyzed by flow cytometry, as described in Methods. The histograms represent profiles of cells from representative mTOR+/+ and mTOR+/kd mice. Percentage CD3 cells out of all cells in a live lymphocyte gate is shown. The data are representative of three pairs of mice examined. Click here for file

Additional file 3 **Normal mTOR activity in livers of mTOR+/kd mice**. mTOR was immunoprecipitated from liver lysates and the immune complex kinase assays were performed with 4E-BP1 as exogenous substrate., as described in Methods. Autoradiogram images showing labeled 4E-BP1 and mTOR are shown. Phoshorylation of 4E-BP1 and of mTOR was quantified using a phosphoimager and normalized to the amount of mTOR present in a kinase reaction (*bottom*). Amount of mTOR in immunoprecipitates or in total lysates was assessed by immunoblotting with mTOR antibody. β-tubulin was used as a loading control. Equal amount of 4E-BP1 in the kinase reaction mixture was visualized by Ponceau S staining. Data are representative of two experiments. Click here for file

Additional file 4 **Normal cell cycle profiles of mTOR+/kd thymocytes**. Isolated thymocytes were stained with propidium idodide for cell cycle analysis via FACS, as described in Methods. The numbers indicate the percentage of cells in G1, S, or G2 cell cycle phases. Click here for file
