# Supplementary figures and images for: A kinase-dead knock-in mutation in mTOR leads to early embryonic lethality and is dispensable for the immune system in heterozygous mice
Source: BMC Immunol. 2009 May 20;10:28. doi: 10.1186/1471-2172-10-28 (PMC2698930; doi:10.1186/1471-2172-10-28)

## Slide 1
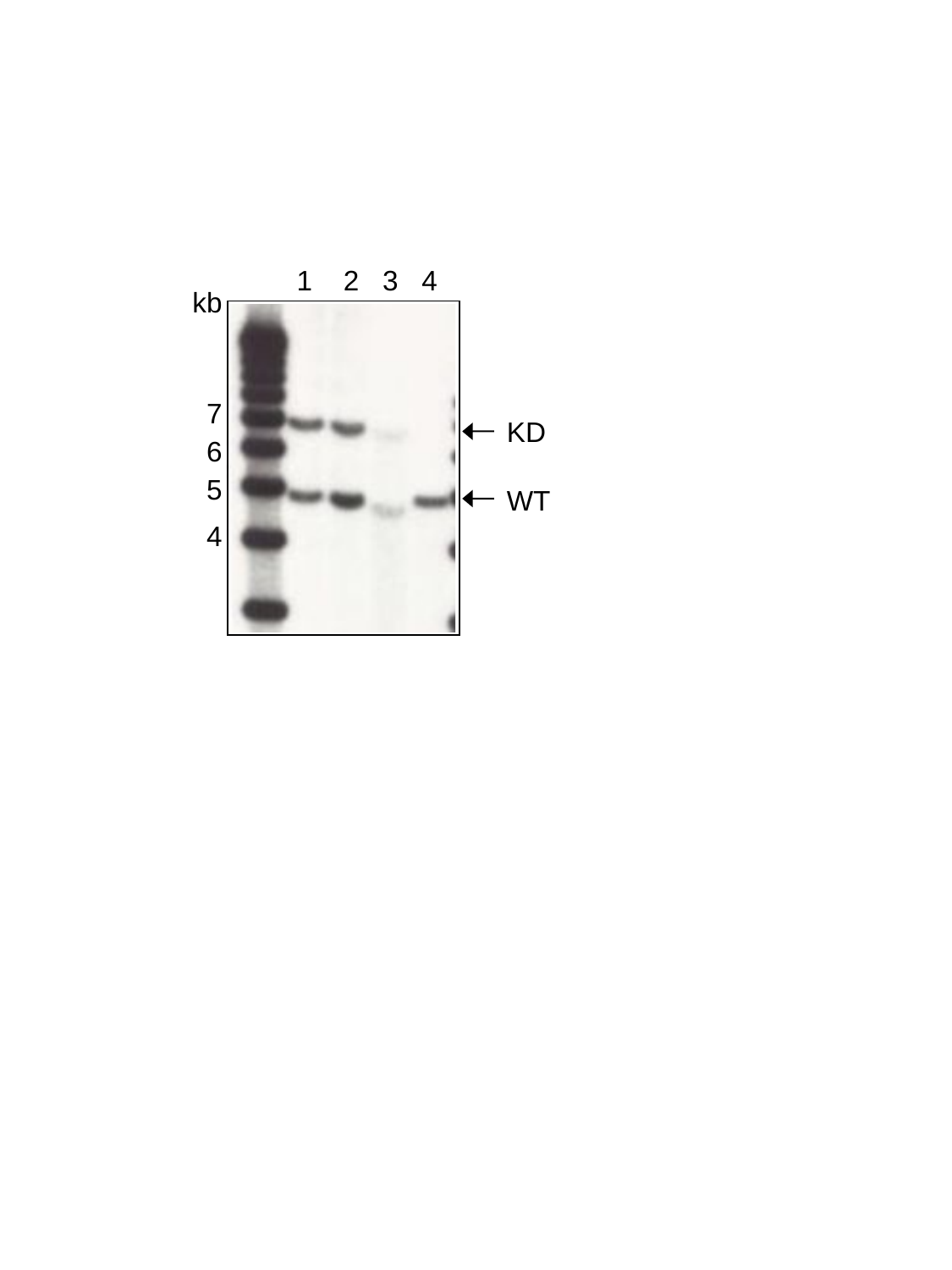

1 2 3 4
kb
7
KD
6
5
WT
4

Supplement: Additional file 1 — Southern blot verification of targeted ES cell clones. Hybridization of Hind III digested genomic DNA from ES cells probed with a 5' external probe shows correct targeting event in lanes#1–3. Lane 4 is a control DNA from parental cells. [file 1471-2172-10-28-S1.ppt]

## Slide 1
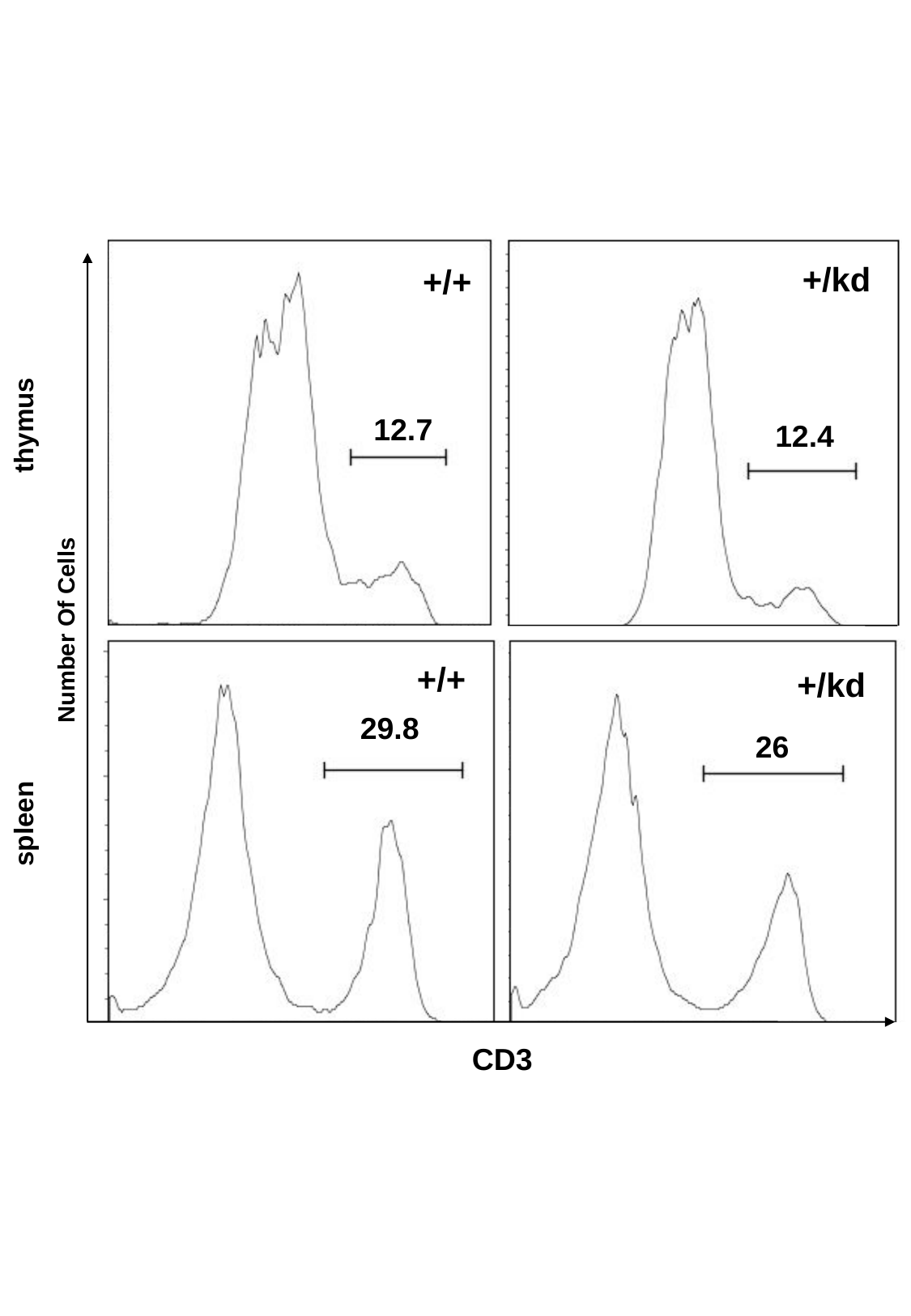

+/kd
12.4
+/+
12.7
thymus
Number Of Cells
+/kd
26
+/+
29.8
spleen
CD3

Supplement: Additional file 2 — CD3 expression in the thymocytes of mTOR+/kd mice. Thymocytes from mTOR+/+ and mTOR+/kd mice were stained for CD3 and analyzed by flow cytometry, as described in Methods. The histograms represent profiles of cells from representative mTOR+/+ and mTOR+/kd mice. Percentage CD3 cells out of all cells in a live lymphocyte gate is shown. The data are representative of three pairs of mice examined. [file 1471-2172-10-28-S2.ppt]

## Slide 1
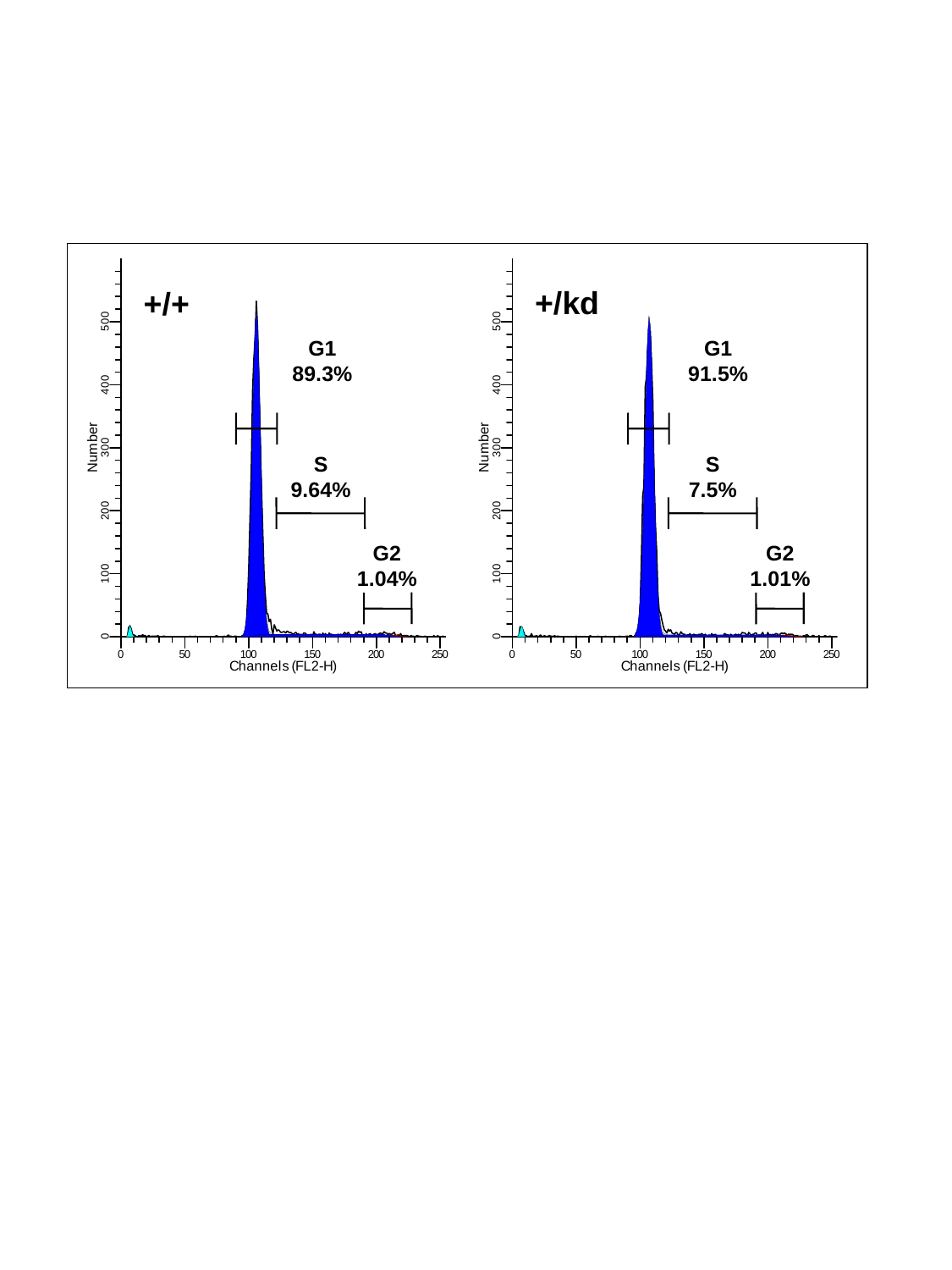

+/kd
+/+
G1
89.3%
G1
91.5%
S
9.64%
S
7.5%
G2
1.04%
G2
1.01%

Supplement: Additional file 4 — Normal cell cycle profiles of mTOR+/kd thymocytes. Isolated thymocytes were stained with propidium idodide for cell cycle analysis via FACS, as described in Methods. The numbers indicate the percentage of cells in G1, S, or G2 cell cycle phases. [file 1471-2172-10-28-S4.ppt]
