# Supplementary material for: A kinase-dead knock-in mutation in mTOR leads to early embryonic lethality and is dispensable for the immune system in heterozygous mice
Source: BMC Immunol. 2009 May 20;10:28. doi: 10.1186/1471-2172-10-28 (PMC2698930; doi:10.1186/1471-2172-10-28)
Supplement: Additional file 3 — Normal mTOR activity in livers of mTOR+/kd mice. mTOR was immunoprecipitated from liver lysates and the immune complex kinase assays were performed with 4E-BP1 as exogenous substrate., as described in Methods. Autoradiogram images showing labeled 4E-BP1 and mTOR are shown. Phoshorylation of 4E-BP1 and of mTOR was quantified using a phosphoimager and normalized to the amount of mTOR present in a kinase reaction (bottom). Amount of mTOR in immunoprecipitates or in total lysates was assessed by immunoblotting with mTOR antibody. β-tubulin was used as a loading control. Equal amount of 4E-BP1 in the kinase reaction mixture was visualized by Ponceau S staining. Data are representative of two experiments. [file 1471-2172-10-28-S3.ppt]

## Slide 1
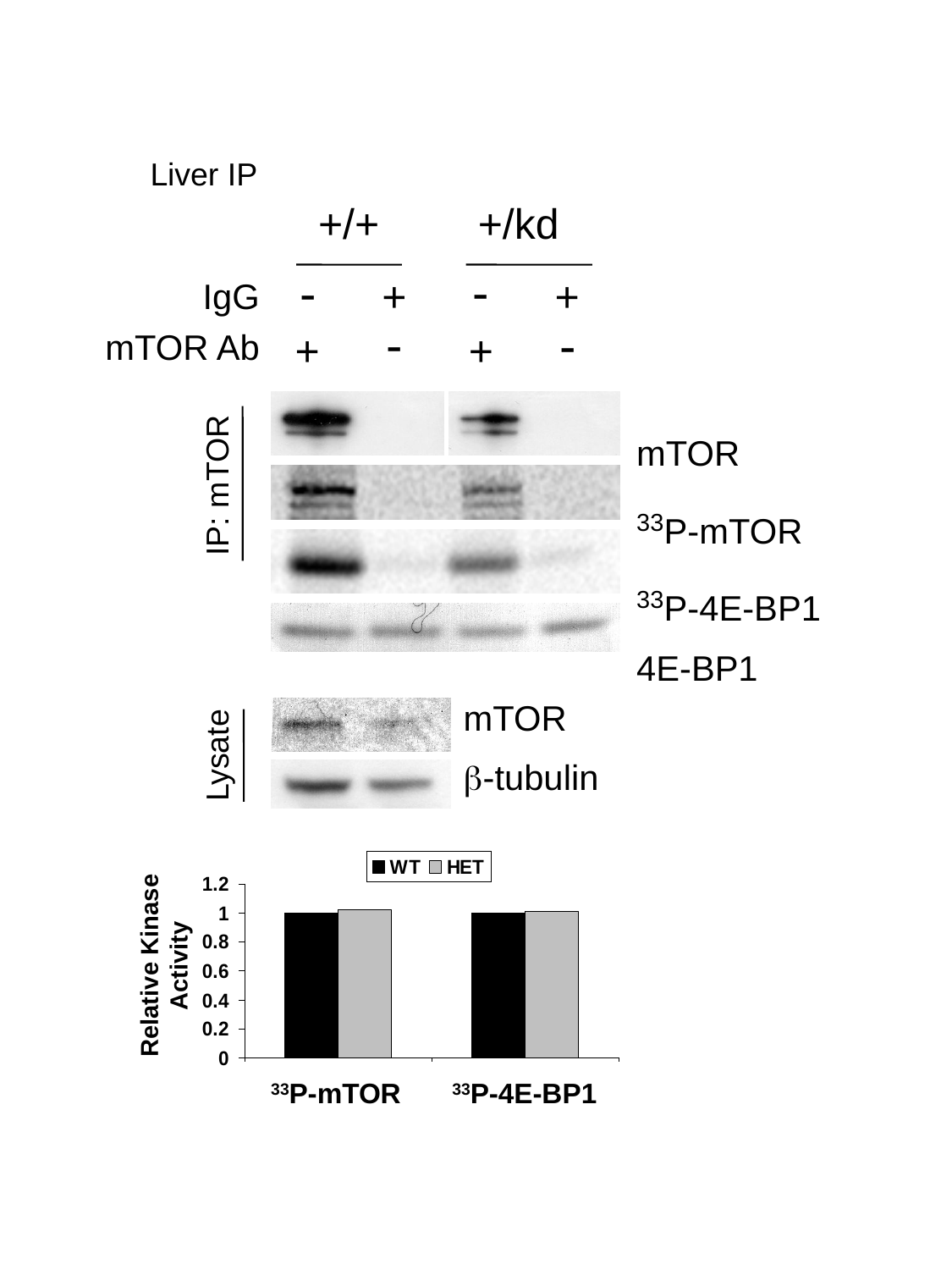

Liver IP
+/+
+/kd
-
-
+
+
IgG
mTOR Ab
-
-
+
+
mTOR
33P-mTOR
33P-4E-BP1
4E-BP1
IP: mTOR
mTOR
β-tubulin
Lysate
Relative Kinase
Activity
33P-mTOR
33P-4E-BP1
